# Maternal Nutrition and Body Composition During Breastfeeding: Association with Human Milk Composition

**DOI:** 10.3390/nu10101379

**Published:** 2018-09-27

**Authors:** Agnieszka Bzikowska-Jura, Aneta Czerwonogrodzka-Senczyna, Gabriela Olędzka, Dorota Szostak-Węgierek, Halina Weker, Aleksandra Wesołowska

**Affiliations:** 1 Department of Clinical Dietetics, Faculty of Health Science, Medical University of Warsaw, E Ciolka Str. 27, 01-445 Warsaw, Poland; agnieszka.bzikowska@wum.edu.pl (A.B.-J.); aneta.senczyna@wum.edu.pl (A.C.-S.); dorota.szostak-wegierek@wum.edu.pl (D.S.-W.); halina.weker@wum.edu.pl (H.W.); 2 Department of Medical Biology, Faculty of Health Science, Medical University of Warsaw, Nowogrodzka Str. 73, 02-018 Warsaw, Poland; 3 Laboratory of Human Milk and Lactation Research at Regional Human Milk Bank in Holy Family Hospital, Faculty of Health Science, Department of Neonatology, Medical University of Warsaw, Zwirki i Wigury Str. 63A, 02-091 Warsaw, Poland; aleksandra.wesolowska@wum.edu.pl

**Keywords:** breastfeeding, human milk composition, body composition, maternal diet

## Abstract

The composition of human milk is dynamic and can vary according to many maternal factors, such as diet and nutritional status. This study investigated the association of maternal nutrition and body composition with human milk composition. All measurements and analyses were done at three time points: during the first (*n* = 40), third (*n* = 22), and sixth (*n* = 15) month of lactation. Human milk was analyzed using the Miris human milk analyzer (HMA), body composition was measured with bioelectrical bioimpedance (BIA) using a Maltron BioScan 920-II, and the assessment of women’s nutrition was based on a three-day dietary record. The correlation coefficient (Pearson’s *r*) did not show a significant statistical relationship between human milk composition and nutrients in women’s diet at three time points. For women in the third month postpartum, we observed moderate to strong significant correlations (*r* ranged from 0.47 to 0.64) between total protein content in milk and the majority of body composition measures as follows: positive correlations: % fat mass (*r* = 0.60; *p* = 0.003), fat-free mass expressed in kg (*r* = 0.63; *p* = 0.001), and muscle mass (*r* = 0.47; *p* = 0.027); and negative correlation: % total body water (*r* = −0.60; *p* = 0.003). The variance in milk fat content was related to the body mass index (BMI), with a significant positive correlation in the first month postpartum (*r* = 0.33; *p* = 0.048). These findings suggest that it is not diet, but rather the maternal body composition that may be associated with the nutritional value of human milk.

## 1. Introduction

Human milk is the best source of nutrition for infants, as it contains essential nutrients in the right balance, and other bioactive factors (e.g., hormones, antibodies, bioactive molecules, stem cells) [1,2]. It is well documented that exclusive breastfeeding for the first six months of life is associated with a decreased incidence of infections and chronic diseases [3,4]. Exclusive breastfeeding has also been shown to trigger a protective effect against later obesity [5,6] and type 2 diabetes in the offspring [7,8]. Nevertheless, this protective effect is controversial, and it may differ in accordance with maternal phenotypes [9,10].

Compared to infant formulas, which have standardized compositions, human milk composition changes dynamically, as it is produced by women with significantly varying genotypes and phenotypes [11]. Table 1 summarizes the milk energy and macronutrient concentration from past studies on different human populations.

**Figure 1:**
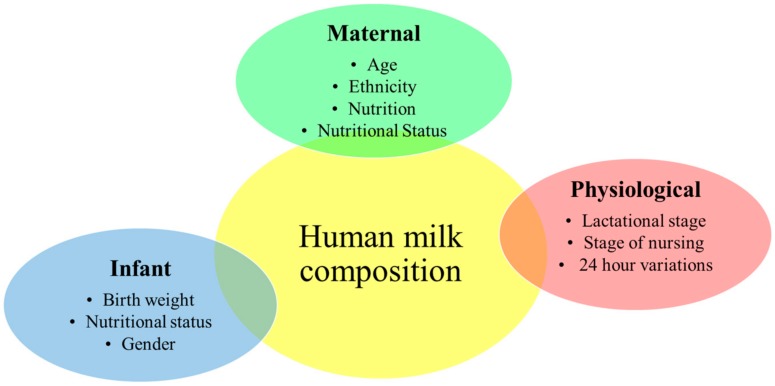
Maternal, infant, and physiological factors that may influence human milk composition.

The composition of human milk is influenced by many maternal, infant, and physiological factors (Figure 1) [11]. Some of these factors are better investigated than others, involving 24 h variations (peak fat content occurs at midmorning) [12], lactational stage (colostrum is reported to be higher in protein but lower in lactose and fat in comparison to mature milk) [13], and time point in breastfeeding session (hindmilk is higher in fat than foremilk; lactose shows an inverse correlation with the stage of breastfeeding) [14,15]. The influence of other factors, including those contained in this study (maternal nutrition and body composition) do not have well-defined effects.

Previous studies assessing the relationship between maternal factors and human milk composition had some limitations, such as no specific time for the expression of milk samples (time of day, hind- or foremilk), and no information about exclusive breastfeeding or analysis of milk composition, excluding nonprotein nitrogen sources, which lead to inflated protein concentrations. Additionally, most of the existing studies were conducted in the 1980s and 1990s [16,17,18] and for milk composition analysis, they used separate analytical instruments for protein, fat, and lactose, which may have been flawed and was time-consuming. All of these limitations influence a true-value assessment of these associations.

We investigated the impact of maternal diet and body composition on human milk composition, analyzing women’s diets and body composition and the nutritional value of human milk.

## 2. Materials and Methods

### 2.1. Study Participants

A convenience sample of breastfeeding women (*n* = 40) was recruited from the community, primarily from the Holy Family Hospital in Warsaw. Participants were enrolled during their first month of lactation. The inclusion criteria were as follows: age ≥ 18 years, full-term delivery (gestational age ≥ 37 weeks), exclusively breastfeeding, and no contraindications to body composition analysis (metal implants, pacemaker, defibrillator, stents, large implants, implanted devices that emit an electronic signal). Exclusion criteria included the following: preexisting chronic or gestational disease, smoking during pregnancy, multiple pregnancy, low birth weight of the newborn, and low milk supply. All mothers provided written informed consent to participate in the study. The study was approved by the Ethics Committee of the Medical University of Warsaw (KB/172/115).

### 2.2. Study Session Design

All measurements and analyses were made at three time points: during the first, third, and sixth months of lactation. Participants visited the Holy Family Hospital in Warsaw. At each study session, the mother was weighed, a body composition analysis was performed, and a 24 h milk collection was taken. Twenty-two women provided milk samples at two time points, and 15 women at three time points. A 3-day dietary record was self-reported by each mother and checked by a qualified dietitian.

### 2.3. Anthropometric Measurements

Body weight and height were measured using a Seca 799 measurement station and column scales (±0.1 kg/cm; Seca, Chino, CA, USA). The body mass index (BMI) was calculated as the ratio between the body weight and the height squared (kg/m^2^). Interpretation of these data followed the classification proposed by the World Health Organization (WHO): below 18.5 kg/m^2^, underweight; 18.5–24.9 kg/m^2^, normal weight; 25.0–29.9 kg/m^2^, pre-obese; ≥30 kg/m^2^, obese [24].

### 2.4. Body Composition Analysis with Bioelectrical Impedance (BIA)

Whole-body impedance (wrist to ankle) of the women was measured using the Maltron BioScan 920-II multifrequency bioelectrical impedance analyzer (Maltron Bioscan, Rayleigh, UK) according to the manufacturer’s instructions [25]. Total body electrical impedance alternated with four frequencies: 5, 50, 100, and 200 kHz. The subjects were measured in a supine position, on a nonconductive surface, after taking a rest for about 10 min. Before the electrodes were placed, the sites were cleaned using isopropyl alcohol to limit possible errors and to ensure adherence. The whole-body impedance vector components resistance (R) and reactance (Xc) were measured at the same time. On this basis, body fat, lean mass, other components, and REE (resting energy expenditure) were calculated. Before taking the BIA measurement, the women were instructed with the following guidelines (according to Heyward and Stolarczyk [26]): no heavy exercise 12 h before the test, no large meals or caffeinated products 4 h before the test, consumption of liquids limited to 1% of body weight or two 8 oz. glasses of water 2 h before the test.

### 2.5. Twenty-Four-Hour Human Milk Collection

Human milk samples were collected by participants at home after they were given detailed instructions on taking, storing, and transporting samples to the Holy Family Hospital in Warsaw. Prefeed and postfeed samples were collected from all participants from all time periods (6:00–12:00, 12:00–18:00, 18:00–24:00, 24:00–6:00) to minimize possible circadian influences on the milk composition. A total of 5–10 mL of prefeed and postfeed milk samples were obtained from the breast(s) the infant fed from, by breast pump, or manually. Samples were stored at −20 °C for later analysis.

### 2.6. Human Milk Composition

Human milk was analyzed using the Miris human milk analyzer (HMA) (Miris, Uppsala, Sweden) with a validated protocol. The HMA is calibrated with human milk standards, and it can measure total and true proteins, fat, lactose, and total solids simultaneously. In addition, the macronutrient content of the milk was used to calculate digestible energy. The concentrations of all macronutrients were reported in grams per 100 mL and energy (kilocalories) was calculated at 4 kcal/g for protein, 9 kcal/g for fat, and 4 kcal/g for carbohydrates. Total protein refers to total nitrogen × 6.25, and true protein is total protein minus 24% for nonprotein nitrogen. Total protein as reported by the Miris analyzer was converted to bioavailable protein (true protein) for the data analysis using the following equation: total protein (grams) × 0.825 [26,27]. The HMA is based on semisolid mid-infrared (MIR) transmission spectroscopy, which is the certified method for milk analysis in the dairy industry according to ISO 9622:1999 by the Association of Official Analytical Chemists (AOAC) and the International Dairy Federation (IDF) [28]. Before analysis, each sample (*n* = 77) was warmed to 40 °C and homogenized for 1.5 s/1 mL of probe using a sonicator (milk homogenizer, Miris, Uppsala, Sweden). From each pool, three samples (~12 mL in total) were taken to analyze the nutritional value, and for the result, we used the average of three measurements.

### 2.7. Nutritional Value of Daily Food Consumption

The assessment of women’s nutrition was based on a 3-day dietary record. Sizes of declared food portions were verified using the “Album of Photographs of Food Products and Dishes” from the National Food and Nutrition Institute [29]. Collected data were used to estimate daily food consumption. Energy and nutritional value of daily food consumption (content of macronutrients, cholesterol, fatty acids, dietary fiber, minerals, and vitamins) were calculated using Dieta 5.0 nutritional software (National Food and Nutrition Institute, Warsaw, Poland).

### 2.8. Statistical Analysis

Statistical analyses were performed using Statistica 12PL, Tulusa, USA and IBM Statistics 21, New York, NY, USA. A *p*-value below 0.05 was adopted as statistically significant. Variable distributions were evaluated with Shapiro–Wilk test, and descriptive statistics (means and standard deviations as well as medians and interquartile ranges) were calculated. The mothers’ anthropometric data and body composition, and the nutritional value of their diet in the first and sixth months of lactation were compared using a paired 2-sample Student’s *t*-test (normal distribution of differences between all pairs), or a Wilcoxon signed-rank test for paired samples (nonnormally distributed differences between all pairs). A trend analysis of milk composition at three time points was performed with the Jonckheere–Terpstra test, and its effect size was estimated with Kendall’s tau-b correlation coefficient. Correlations between milk composition and the mothers’ body composition and diet were estimated with Pearson’s r correlation coefficient.

## 3. Results

### 3.1. Subjects and Human Milk Composition

All participants had a university education and a high socioeconomic status. The subjects’ anthropometric data and body composition measures are shown in Table 2. The mean maternal age was 31.1 ± 4.4 years. At the first month postpartum, none of the participants were classified as being underweight (BMI < 18.5 kg/m^2^). Most of them (*n* = 32, 80%) had normal body mass, and 20% (*n* = 8) were overweight. We did not observe any statistically significant differences between maternal body composition at the first and third months postpartum. However, there were statistically significant differences between weight and BMI values in the first and sixth months of lactation. The Wilcoxon signed-rank test was 2.59, *p* = 0.009 for weight and 2.67, *p* = 0.008 for BMI.

Table 3 provides the average results of the nutritional value of human milk (energy, macronutrients, and dry matter) and changes in the concentrations of the components at three time points. We observed a statistically significant downward trend for total protein (tau-b = −0.31; *p* = 0.001) and true protein (tau-b = −0.30; *p* = 0.001) concentration in human milk. We also noted a decreasing energy value, but the trend was not statistically significant (tau-b = −0.18; *p* = 0.052).

### 3.2. Nutritional Value of Daily Food Consumption

Table 4 presents the results of energy and nutrient intake in relation to Polish nutritional standards [29]. The risk of deficient energy intake was observed in 100% of the women at three time points. There were no significant differences between the intake of macronutrients (protein, fat, and carbohydrates), minerals, and vitamins at each stage of the study. Among all of the women (100%), at every time point we observed an insufficient intake of vitamin D. The majority of women (60%, *n* = 24) at the first month postpartum did not reach the estimated average requirement (EAR) value for calcium.

### 3.3. Association between Maternal Diet and Milk Composition

Table 5 presents correlation coefficient (Pearson’s *r*) between human milk composition and nutrients in the mother’s diet at three time points (first, third, and sixth month of lactation). We did not observe any statistically significant correlation between these factors (*p* > 0.05). The estimated energy value and the macronutrient content in lactating women’s average daily food consumption recorded for three days did not allow for the prediction of the variance in their milk composition.

### 3.4. Association between Maternal Body Composition and Milk Composition

Table 6 presents the correlation coefficient (Pearson’s *r*) between human milk composition and lactating mothers’ body composition at three time points (first, third, and sixth month of lactation).

For women in the third month postpartum, we observed moderate to strong significant correlations (*r* ranged from 0.45 to 0.52) between the true protein content in their milk and the majority of body composition measures. In the third month of lactation, the total protein in milk correlated positively with the mothers’ weight (*r* = 0.63; *p* = 0.002), BMI (*r* = 0.59; *p* = 0.004), % fat mass (*r* = 0.60; *p* = 0.003), fat-free mass expressed in kg (*r* = 0.63; *p* = 0.001), and muscle mass (*r* = 0.47; *p* = 0.027) and negatively with percentage of total body water (*r* = −0.60; *p* = 0.003).

The variance in milk fat content was related to the lactating women’s weight, with a significant positive correlation in the sixth month postpartum (*r* = 0.49; *p* = 0.039), with BMI, and a significant positive correlation in the first month postpartum (*r* = 0.33; *p* = 0.048).

Similar to the protein concentration, the energy value of human milk was highly correlated with the maternal body composition. In the third month postpartum, we found positive correlations with weight (*r* = 0.43; *p* = 0.048), BMI (*r* = 0.39; *p* = 0.049), and muscle mass (*r* = 0.44; *p* = 0.041). There was a negative correlation with % total body water (*r* = −0.60; *p* = 0.032) in the third month of lactation.

We did not observe significant correlations between the carbohydrate content in human milk and measures of the body composition of lactating women, except for a positive correlation with the percentage of intracellular water and a negative correlation with the percentage of extracellular water in the third month postpartum.

## 4. Discussion

In our study, we found that protein (total and true) and carbohydrate concentrations in human milk were significantly different, depending on the period of lactation. From the first to the sixth month of lactation, total and true protein concentrations significantly decreased. We did not find any relationships between the nutritional value of maternal daily food consumption and milk composition. Maternal BMI and adiposity were positively associated with the protein content of milk.

The total milk protein content in our study was high compared to mature milk from Chinese (0.9 g/100 mL [19]), Brazilian (1.1 g/100 mL [22]), and Australian mothers (1.0 g/100 mL [23]). The analysis of carbohydrates by HMA MIRIS in human milk is affected by the presence of lactose and nonlactose carbohydrates, primarily human milk oligosaccharides (HMOs) [27]. Some of the divergence between the findings for lactose concentrations may be related to the inclusion of HMOs in the mid-infrared (mid-IR) transmission spectroscopy measurements [28]. Since the reference laboratory analysis for lactose concentration, high-pressure liquid chromatography (HPLC), does not measure HMOs, it is probable that lactose levels measured by mid-infrared transmission spectroscopy were a result of absorbing terminal or core lactose moieties of HMOs [30]. The measured concentration of carbohydrates in our study (7.0–7.1 g/100 mL) was consistent with the normal range in human milk [31]; however, it has been reported that lactose concentration can vary from 6.3 to 8.1 g/100 mL [32]. The reasons for this variability may relate to the time point in the breastfeeding session (pre- or postfeeding), the time of feeding, feeding frequency, or the milk analysis method [33]. Fat is known to be the most variable macronutrient in human milk. In the first month postpartum, the median fat concentration in milk was 3.5 g/100 mL. Our finding was consistent with those in Japan (3.6 g/100 mL) [19], China (3.4 g/100 mL) [34], and the United States (3.6 g/100 mL) [35].

It is reported that sampling protocols are of prime importance when investigating the association between human milk composition and maternal factors. The direct relationship between the dietary intake of single nutrients and their presence within human milk is hard to study for many reasons. These include difficulties in collecting nutrition data and the availability of reliable human milk samples. In the present study, the procedure of milk collection (using a 24-h period) was performed to minimize errors. We also confirmed a systemic change between fore- and hindmilk samples for concentrations of energy and macronutrients. Additionally, all of the milk samples were from mothers practicing exclusive breastfeeding.

We found no evidence for associations between the maternal intake of any dietary nutrients and the milk composition in this sample. This is consistent with past studies, which showed that milk nutrient composition appears to be mainly independent of the nutritional value of maternal daily food consumption [36]. This absence of an effect of maternal diet is evident in both observational and experimental studies, in which nutritional supplements were shown to result in minimal or trifling changes to milk macronutrient content [21,35]. Some compensatory physiological mechanisms might be responsible for the comparatively stable milk macronutrient composition related to the nutritional variations of maternal diet [33]. Tigas et al. [37] reported that increased glucose demands during lactation are met by increased glucose production as a result of increased glycogenolysis, but not gluconeogenesis, or by an increased use of free fatty acids. These results are consistent with the hypothesis that human milk composition might be buffered against variations in the maternal dietary intake of each component [15]. For instance, Rakicioğlu et al. [38] found that short-term fasting or dieting by lactating women has not been associated with milk composition, despite the fact that the nutritional status of lactating women was affected by Ramadan fasting, when all macronutrient intake decreased.

The concentration of lactose in human milk is known to be the least variable of the macronutrients [32]. No significant relationships were found between milk lactose and a maternal diet high in fat and low in carbohydrates, compared with a diet low in fat and high in carbohydrates [39]. Additionally, there were no significant differences between milk lactose and a high-protein diet [40], or between vegetarian and non-vegetarian diets [41]. Considering the total protein concentration, studies from Europe and the United States did not report any relationship between milk total protein and maternal intake of animal and vegetable protein [42]. The variation of total fat concentration in human milk also appears to be independent of maternal diet [30]. Nevertheless, the specific fatty acids that form the total lipid fraction are sensitive to maternal nutrition. These fatty acids are either taken up from the maternal plasma, or synthetized endogenously by the mammary glands. Both of these sources are influenced by maternal diet composition [37,43,44,45].

While there is a lack of a relationship between milk composition and maternal nutrition, we observed that concentrations of several nutrients in milk were correlated with maternal body composition, depending on the postpartum period.

Previous studies based on maternal BMI reported a positive relationship with fat concentration in human milk [13,19,45], which is consistent with our results. We found that in the first month postpartum, maternal BMI was correlated with milk fat content (0.33; *p* = 0.048). Chang et al. [13] reported that the mother’s current BMI was positively correlated with lipid levels at 1–2 weeks (0.151; *p* < 0.05), 2–3 months (0.151; *p* < 0.05), and 7–8 months (0.153; *p* < 0.05). Contrary to these findings, Bachour et al. [46] suggested that there were no associations between maternal body mass index and fat concentration in human milk. Interestingly, Quinn et al. [21] observed that women in Cebu with lower BMI tended to produce milk with higher fat contents than women with higher BMI. The significant inverse association between milk fat and BMI suggests that the sample BMI was indexing lean mass. In our study, we found no evidence for an association between maternal body composition and fat concentration in milk at any time point. Although past research has at times reported a relationship between maternal fat mass and milk fat content, these associations were often limited to overweight or obese women [47,48]. It has been also suggested that increasing maternal adiposity may be related to impaired milk sugar synthesis, and that the lipid increase reflects this decrease in lactose [49].

A few studies investigating the associations between human milk protein concentration and maternal nutritional status are contradictory, with some reporting a positive relationship between protein and maternal adiposity as assessed by BMI [13,18,45,50] and one a negative association between total protein content and maternal BMI [21]. However, it must be stressed that BMI is not a direct measure of adiposity, so that the strength of the relationship between protein concentration and BMI may not reflect the true value of these associations. Kugananthan et al. [51] and Quinn et al. [52] observed that a higher maternal fat mass percentage, but not BMI, was associated with higher protein concentrations in milk. Using advanced techniques to evaluate maternal body composition, we found that maternal body composition was highly correlated with total protein concentration in milk. We reported a positive correlation with maternal weight (*p* = 0.002), BMI (*p* = 0.004), % fat mass (*p* = 0.003), and muscles (*p* = 0.027), and a negative correlation with % total body water (*p* = 0.003) in the third month of lactation. A decreased total body water content is characteristic for women with more adipose tissue, explaining that negative correlation. It is also reported that several serum amino acid concentrations, in particular branched-chain amino acids (BCAAs), are increased in mothers with more adipose tissue [53], leading to more amino acids transferred to the breast and milk [54]. This may explain the positive relationship between maternal adiposity and milk protein concentration [18].

The concentration of carbohydrates in human milk is the least variable of the macronutrients. Considering that a stable concentration of lactose is important for maintaining a constant osmotic pressure in milk [55], maternal nutritional status is not expected to have a meaningful impact on total carbohydrate concentrations in milk. In our study, the measured concentrations of carbohydrates were not related to maternal BMI and body composition at any time point. This is in line with a previous study carried out by Kugananthan et al. [51], which showed that that the lactose concentration in human milk (measured by enzymatic spectrophotometric method) was not related to maternal adiposity profiles (BMI, *p* = 0.66; % fat mass, *p* = 0.48). By contrast, Chang et al. [13] reported that maternal BMI was negatively correlated with lactose concentrations at 4–5 months (0.148; *p* < 0.05) and 6–7 months (0.242; *p* < 0.01) postpartum.

The strengths of this study are the use of advanced techniques to assess maternal body composition and the milk collection protocol, which allowed possible errors in human milk composition to be minimized. The limitations of this study are convenience sampling, the modest number of participants, mainly at six months postpartum, resulting from discontinuation of breastfeeding, and the constraints associated with multiple measurement time points. BIA analysis incorporates various assumptions, and it may also result in less precise estimates, mainly in situations in which the water–electrolyte balance is altered. If used to monitor individuals over time, it can indicate the direction, but not the magnitude of changes in lean mass. Further, our population was Caucasian, with university educations and a high socioeconomic status. All indicated limitations decreased the representativity of the study, and caution should be used when extrapolating the results.

Considering that human milk provides not only energy and nutrients, but also bioactive factors that are crucial for infant growth and development, human milk composition research should continue in order to identify factors that may be associated with changes in its composition. All of these efforts may contribute to accomplishing optimum growth, development, and health in infants.

## Figures and Tables

**Table 1:** Variations in mature human milk composition in different sample populations.

Population	Characteristics of Participants	Analytical Technique	Energy ^1^	Fat ^2^	Lactose ^2^	Protein ^2^	Reference
			Mean ± SD				
Korea	2632 healthy lactating mothers of full-term infants (32.0 ± 3.3 years)	Infrared spectrophotometry (MilkoScan FT2)	61.1 ± 12.1	3.0 ± 1.4	7.1 ± 0.4	1.4 ± 0.3	Chang, 2015 [13]
China	436 urban Chinese lactating mothers	Infrared spectrophotometry	61.3	3.4	7.1	0.9	Yang, 2014 [19]
Tibet	82 breastfeeding Tibetans living at high and low altitudes in rural villages	Micro Rose–Gottlieb	81.4 ± 17.4	5.3 ± 2.0	7.4 ± 0.5	1.3 ± 0.4	Quinn, 2016 [20]
Philippines	102 Filipino breastfeeding mothers (24.6–25.4 years)	Micro Rose–Gottlieb	68.6 ± 15.0	3.8 ± 1.5	7.3 ± 0.6	1.3 ± 0.5	Quinn, 2012 [21]
Brazil	34 donors of the Human Milk Bank	Infrared spectrophotometry (MilkoScan Minor)	56.7 ± 11.7	3.1± 1.18	6.1 ± 0.6	1.22 ± 0.5	Abranches, 2014 [22]
United States (DARLING study)	58 healthy lactating mothers planning to continue nursing for a minimum of 12 months	Lowry assay (protein), Folch extraction (fat), colorimetric (lactose)	69.7 ± 6.7	3.6 ± 0.7	7.4 ± 0.15	1.2 ± 0.15	Nommsen, 1991 [18]
Australia	23 lactating mothers of healthy term infants (33.0 ± 3 years)	Mid-infrared spectrophotometry (MIRIS)	82.4	5.9	6.3	1.0	Khan, 2013 [23]

^1^ Energy is presented as kilocalories (kcal) per 100 mL. ^2^ Macronutrients are presented as grams per 100 mL. SD, standard deviation.

**Table 2:** Subjects’ anthropometric data and body composition measures.

Characteristic	Month of Lactation			Difference between First and Sixth Month of Lactation (*p*-Value)
	1 (*n* = 40)	3 (*n* = 22)	6 (*n* = 15)	
Weight (kg)	64.5 ± 12.2 62.3 (54.8–70.9)	65.1 ± 13.2 62.4 (54.7–70.5)	61.4 ± 10.0 59.5 (54.6–67.0)	0.009 ^2,^*
Height (cm)	166.6 ± 6.6 166.5 (162.0–172.5)	166.6 ± 6.6 166.5 (162.0–172.5)	166.6 ± 6.6 166.5 (162.0–172.5)	–
Body mass index (kg/m^2^)	23.0 ± 3.6 22.7 (20.4–24.8)	23.3 ± 4.0 23.0 (19.9–25.9)	21.8 ± 2.8 21.5 (19.8–23.7)	0.008 ^2,^*
Fat mass (kg)	19.8 ± 10.3 17.9 (11.3–23.0)	19.8 ± 9.5 19.3 (12.6–23.3)	16.9 ± 6.6 17.7 (9.8–21.3)	0.064 ^2^
Fat mass (%)	28.2 ± 8.5 28.5 (20.6–33.0)	28.9 ± 8.6 30.2 (21.3–34.6)	26.4 ± 7.1 26.7 (19.2–32.4)	0.174 ^1^
Fat-free mass (kg)	45.4 ± 3.9 45.7 (43.0–48.4)	45.3 ± 4.6 45.1 (40.9–49.2)	45.0 ± 4.4 43.5 (40.9–49.4)	0.701 ^1^
Total body water (L)	32.4 ± 3.8 31.2 (29.4–35.2)	32.2 ± 4.1 31.0 (28.5–34.7)	31.5 ± 3.7 30.6 (28.5–34.1)	0.695 ^2^
Total body water (%)	51.2 ± 5.1 50.3 (46.9–55.3)	50.3 ± 4.9 49.8 (46.5–52.2)	51.2 ± 4.1 50.9 (46.9–54.8)	0.236 ^1^
Protein (kg)	9.0 ± 1.4 9.0 (8.5–9.9)	9.3 ± 1.3 9.2 (8.4–10.6)	9.6 ± 1.0 9.4 (8.7–10.6)	0.638 ^2^
Muscles (kg)	19.9 ± 1.9 19.8 (18.8–21.4)	19.8 ± 2.0 19.7 (18.3–21.7)	19.3 ± 1.7 18.9 (18.3–21.5)	0.084 ^2^

Data are mean ± standard deviation (SD), median, and ranges. ^1^ Paired two-sample Student’s *t*-test; ^2^ Wilcoxon signed rank test for paired samples. * *p* < 0.05.

**Table 3:** Composition of human milk.

	Month of Lactation			Linear Trend (*p*-Value)	Effect Size (Kendall’s Tau-b)
	1 (*n* = 40)	3 (*n* = 22)	6 (*n* = 15)		
Energy (kcal/100 mL)	65.9 ± 9.9 65.5 (62.0–72.5)	61.8 ± 14.4 61.3 (49.0–71.7)	61.2 ± 14.2 60.0 (47.0–73.7)	0.052	−0.18
Fat (g/100 mL)	3.5 ± 1.0 3.5 (3.1–4.3)	3.2 ± 1.5 3.1 (1.8–4.1)	3.2 ± 1.4 3.1 (1.8–4.5)	0.120	−0.14
Total protein (g/100 mL)	1.2 ± 0.2 1.1 (1.1–1.2)	1.1 ± 0.1 1.0 (1.0–1.1)	1.0 ± 0.3 1.0 (0.9–1.1)	0.001	−0.31
True protein (g/100 mL)	0.9 ± 0.2 0.9 (0.8–1.0)	0.8 ± 0.1 0.8 (0.7–0.9)	0.8 ± 0.2 0.7 (0.7–0.9)	0.001	−0.30
Carbohydrates (g/100 mL)	7.0 ± 0.3 7.0 (6.8–7.2)	7.0 ± 0.4 7.1 (6.9–7.2)	7.1 ± 0.4 7.1 (6.9–7.4)	0.236	0.11
Dry matter (g/100 mL)	11.7 ± 1.3 11.8 (11.2–12.7)	11.4 ± 1.7 11.3 (9.9–12.6)	11.4 ± 1.8 11.0 (9.7–12.9)	0.298	−0.09

**Table 4:** Energy and nutrient intake in relation to Polish nutritional standards 2012 [29].

	Month of Lactation			Difference between First and Sixth Month of Lactation (*p*-Value)	Nutritional Standards EAR/RDA/AI
	1 (*n* = 40)	3 (*n* = 22)	6 (*n* = 15)		
Energy (kcal)	1822.7 ± 445.7 1798.4 (1504.4–2070.8)	1825.3 ± 462.0 1773.4 (1508.3–2052.2)	1614.6 ± 435.0 1487.3 (1343.3–1957.7)	0.295 *	2555
Protein (g)	77.7 ± 21.4 76.5 (61.0–91.4)	74.9 ± 19.2 74.9 (64.9–86.6)	66.2 ± 19.9 60.2 (53.3–91.0)	0.187 *	1.17 g/kg weight
Protein (% kcal)	17.3 ± 3.3 17.8 (14.6–19.5)	16.7 ± 2.7 16.3 (14.3–18.8)	16.6 ± 3.0 16.4 (14.7–19.2)	0.567 *	15
Fat (g)	63.4 ± 20.0 62.7 (49.3–74.7)	66.3 ± 23.5 62.0 (50.1–72.1)	55.8 ± 22.8 50.8 (40.2–66.6)	0.805 *	90
SFA	23.6 ± 10.1 20.5 (17.9–28.0)	22.8 ± 12.2 21.0 (18.1–25.8)	20.6 ± 7.4 19.2 (15.4–22.5)	0.846 *	–
MUFA	24.04 ± 8.8 23.8 (16.2–27.8)	24.7 ± 8.6 22.9 (20.5–25.9)	21.4 ± 11.1 18.1 (14.2–25.0)	0.989 *	–
PUFA	10.8 ± 4.4 10.3 (7.1–12.7)	13.8 ± 10.4 11.6 (7.2–15.2)	9.3 ± 9.0 6.7 (4.7–8.1)	0.173 **	–
Fat (% kcal)	30.8 ± 5.8 30.2 (26.4–34.4)	31.9 ± 5.3 30.3 (27.93–36.4)	30.4 ± 7.7 30.0 (23.8–35.9)	0.585 *	30
Cholesterol (mg)	265.7 ± 112.4 253.6 (211.5–339.7)	247.7 ± 135.8 219.0 (165.8–362.3)	255.1 ± 139.6 226.4 (139.2–339.3)	0.879 *	–
Carbohydrates (g)	255.3 ± 65.8 253.5 (204.0–297.0)	253.1 ± 62.4 234.0 (208.8–295.2)	229.7 ± 66.3 247.1 (176.2–290.2)	0.217 *	>175
Carbohydrates (% kcal)	51.9 ± 6.6 51.4 (48.1–56.3)	51.4 ± 5.3 50.7 (48.0–56.2)	53.0 ± 9.2 51.6 (46.3–57.6)	0.863 *	45–65
Sucrose (g)	50.4 ± 30.2 42.6 (28.5–69.1)	46.4 ± 32.7 36.1 (25.5–55.0)	48.7 ± 30.4 44.6 (28.0–58.5)	0.687 *	–
Dietary fiber (g)	21.8 ± 7.2 20.7 (172–25.6)	22.7 ± 9.3 21.6 (16.2–29.2)	19.3 ± 7.6 19.0 (14.5–23.7)	0.251 *	–
Sodium (mg)	2612.4 ± 881.6 2588.0 (2036.7–3179.6)	2359.4 ± 804.3 2280.3 (2064.2–2918.6)	2314.2 ± 745.3 2139.1 (1757.5–2663.9)	0.955 *	1500
Potassium (mg)	3132.6 ± 881.6 2923.7 (2512.5–3576.2)	3009.9 ± 745.5 2894.8 (2655.8–3497.3)	2953.8 ± 846.8 2770.0 (25340.0–3532.4)	0.392 *	4000
Calcium (mg)	745.9 ± 347.3 710.0 (548.2–927.3)	613.3 ± 256.1 598.9 (421.9–774.0)	659.0 ± 302.0 566.1 (459.0–897.2)	0.812 *	800.0
Phosphorus (mg)	1326.7 ± 350.2 1255.5 (1046.9–1555.3)	1320.6 ± 314.1 1347.0 (1204.9–1473.0)	1146.5 ± 383.8 1005.3 (824.6–1441.6)	0.234 *	580
Magnesium (mg)	322.1 ± 87.7 305.8 (263.2–363.8)	351.7 ± 129.2 344.5 (258.9–407.2)	315.5 ± 133.8 283.2 (226.1–406.8)	0.820 *	265.0
Iron (mg)	12.8 ± 9.4 11.0 (9.3–13.3)	12.2 ± 3.7 11.6 (9.6–15.1)	12.6 ± 8.7 10.6 (7.9–13.3)	0.955 **	7.0
Zinc (mg)	10.5 ± 3.9 9.8 (8.9–11.0)	10.2 ± 2.2 9.9 (9.5–11.2)	8.6 ± 2.6 7.8 (6.5–10.9)	0.132 *	10.4
Iodine (µg)	106.0 ± 35.6 102.8 (86.2–128.4)	90.8 ± 37.6 87.2 (64.9–111.2)	88.7 ± 35.2 84.6 (58.6–110.5)	0.656 *	210.0
Vitamin A (µg)	1216.1 ± 680.4 1023.9 (838.3–1297.5)	1390.0 ± 1392.0 960.9 (628.6–1701.0)	1049.3 ± 435.4 893.9 (692.4–1539.9)	0.496 **	900
Vitamin D (µg)	3.23 ± 2.6 2.3 (1.5–3.7)	3.2 ± 2.7 1.9 (1.3–5.0)	2.7 ± 2.3 1.6 (1.2–4.6)	0.874 *	15.0
Vitamin E (mg)	10.4 ± 4.6 9.4 (6.8–13.6)	11.9 ± 7.9 9.3 (7.2–16.0)	10.4 ± 6.8 7.9 (6.5–14.8)	0.873 *	11.0
Vitamin B_1_ (mg)	1.2 ± 0.4 1.2 (1.0–1.4)	1.4 ± 0.5 1.2 (1.0–1.7)	1.2 ± 0.5 1.1 (0.9–1.5)	0.825 *	1.3
Vitamin B_2_ (mg)	1.8 ± 0.6 1.6 (1.3–2.1)	1.6 ± 0.5 1.6 (1.2–1.9)	1.7 ± 0.6 1.5 (1.2–2.1)	0.920 *	1.3
Vitamin PP (mg)	16.2 ± 6.2 14.9 (11.0–18.5)	16.8 ± 6.0 17.8 (11.9–20.3)	16.6 ± 8.8 13.0 (11.0–20.3)	0.936 *	13.0
Vitamin B_6_ (mg)	1.9 ± 0.7 1.8 (1.4–2.2)	1.9 ± 0.6 1.9 (1.4–2.2)	4.8 ± 8.3 1.9 (1.3–2.6)	1.00 **	1.7
Vitamin C (mg)	127.8 ± 109.1 92.3 (57.6–139.9)	135.6 ± 83.9 113.8 (79.1–160.7)	163.0 ± 81.2 139.8 (120.7–253.7)	0.730 *	100
Vitamin B_12_ (µg)	3.75 ± 1.7 3.4 (2.3–4.8)	4.04 ± 3.1 3.0 (1.9–4.9)	3.2 ± 1.8 2.9 (1.9–4.1)	0.900 *	2.4
Folic acid (µg)	324.6 ± 141.2 (239.2–379.3)	310.0 ± 109.9 288.4 (239.5–388.6)	436.5 ± 213.7 364.6 (284.6–672.8)	0.099 *	450.0

Data are mean ± standard deviation (SD), median, and ranges. * Paired two-sample Student’s *t*-test; ** Wilcoxon signed rank test for paired samples. SFA, saturated fatty acids; MUFA, monounsaturated fatty acids; PUFA, polyunsaturated fatty acids; EAR, estimated average requirement; RDA, recommended daily allowance; AI, adequate intake.

**Table 5:** Correlations between human milk composition and nutrients in mothers’ diet.

Energy and Nutrients in Mothers’ Diet	Month of Lactation	Composition of Human Milk					
		Energy ^1^	Total Protein ^2^	True Protein ^2^	Fat ^2^	Carbohydrates ^2^	Dry Matter ^2^
Energy (kcal)	1	0.06	0.08	0.13	0.08	−0.14	0.02
	3	0.12	−0.05	0.02	0.11	0.08	0.11
	6	−0.15	−0.12	−0.05	−0.09	−0.22	−0.11
Protein (g)	1	0.08	0.02	0.02	0.08	−0.05	0.06
	3	0.10	−0.19	−0.12	−0.13	−0.20	0.05
	6	0.01	−0.7	−0.05	0.05	−0.24	0.03
Fat (g)	1	0.09	0.01	0.04	0.08	−0.02	0.11
	3	0.09	0.07	0.10	0.06	0.15	0.10
	6	−0.01	−0.12	−0.03	0.03	−0.10	0.05
Carbohydrates (g)	1	0.01	0.13	0.20	0.05	−0.22	−0.09
	3	0.15	−0.06	0.04	0.14	0.06	0.13
	6	−0.27	−0.08	−0.03	−0.21	−0.26	−0.25
Percent of energy from protein	1	0.02	−0.06	−0.13	−0.01	0.12	0.06
	3	−0.01	−0.18	−0.16	0.05	−0.42	−0.06
	6	0.25	0.13	0.05	0.24	−0.01	0.25
Percent of energy from fat	1	0.13	−0.07	−0.09	0.09	0.21	0.24
	3	0.01	0.16	0.14	−0.02	0.22	0.05
	6	0.23	−0.08	−0.04	0.22	0.13	0.27
Percent of energy from carbohydrates	1	−0.13	0.09	0.15	−0.08	−0.24	−0.24
	3	−0.01	−0.06	−0.06	−0.01	0.01	−0.02
	6	−0.27	0.03	0.02	−0.26	−0.10	−0.30
Sodium (mg)	1	0.01	0.20	0.25	−0.03	−0.01	0.08
	3	−0.22	0.02	0.05	−0.22	−0.18	−0.24
	6	−0.17	0.33	0.40	−0.18	0.01	−0.19
Potassium (mg)	1	0.09	−0.02	0.04	0.11	−0.04	−0.05
	3	0.21	−0.01	0.20	0.22	−0.07	0.18
	6	−0.37	−0.03	0.04	−0.34	−0.48	−0.35
Calcium (mg)	1	0.32	0.08	0.08	0.30	0.21	0.29
	3	0.21	−0.20	−0.23	0.19	0.26	0.23
	6	0.11	−0.12	−0.18	0.20	−0.24	0.13
Phosphorus (mg)	1	0.12	−0.03	−0.03	0.13	−0.01	0.09
	3	0.19	0.02	0.08	0.19	−0.06	0.17
	6	−0.04	−0.05	−0.02	0.02	−0.40	−0.02
Magnesium (mg)	1	0.04	−0.04	−0.02	0.03	−0.02	−0.11
	3	0.27	0.26	0.37	0.27	0.00	0.27
	6	−0.34	0.01	0.06	−0.37	−0.32	−0.33
Iron (mg)	1	−0.04	−0.06	−0.01	−0.02	−0.11	−0.13
	3	0.26	0.24	0.43	0.26	−0.01	0.24
	6	0.01	0.10	0.17	−0.04	0.08	0.03
Zinc (mg)	1	−0.06	−0.06	−0.02	−0.05	−0.10	−0.06
	3	0.26	0.24	0.43	0.26	−0.01	0.24
	6	−0.01	0.13	0.20	0.02	−0.24	0.02
Iodine (µg)	1	0.10	0.20	0.18	0.08	0.01	−0.03
	3	0.10	−0.28	−0.24	0.14	−0.21	0.04
	6	−0.12	0.10	0.14	−0.12	−0.18	−0.20
Vitamin A (µg)	1	−0.17	−0.01	0.05	−0.23	−0.07	−0.21
	3	−0.21	−0.10	0.12	−0.20	−0.22	−0.26
	6	−0.14	−0.16	−0.25	−0.13	−0.14	−0.17
Vitamin D (µg)	1	−0.09	−0.09	−0.08	−0.15	−0.09	−0.14
	3	0.23	0.16	0.18	0.28	−0.41	0.16
	6	0.33	0.06	0.08	0.35	0.03	0.33
Vitamin E (mg)	1	0.20	0.02	0.10	0.22	0.06	0.09
	3	0.19	0.06	0.27	0.20	−0.02	0.17
	6	−0.30	−0.02	0.13	−0.34	−0.36	−0.29
Vitamin B_1_ (mg)	1	−0.21	−0.14	−0.09	−0.19	−0.22	−0.36
	3	0.12	0.19	0.30	0.14	−0.21	0.08
	6	−0.08	−0.07	−0.01	−0.07	0.07	−0.01
Vitamin B_2_ (mg)	1	0.15	−0.02	−0.02	0.18	0.08	0.09
	3	0.08	−0.18	0.02	0.10	−0.10	0.05
	6	0.17	−0.01	−0.02	0.21	−0.04	0.20
Vitamin PP (mg)	1	−0.20	−0.18	−0.15	−0.18	−0.30	−0.24
	3	0.14	−0.02	0.10	0.18	−0.25	0.09
	6	−0.27	0.02	0.05	−0.30	−0.08	−0.19
Vitamin B_6_ (mg)	1	−0.08	−0.10	−0.05	−0.07	−0.14	−0.24
	3	0.10	0.02	0.21	0.13	−0.26	0.04
	6	−0.06	−0.00	−0.07	−0.09	0.16	−0.07
Vitamin C (mg)	1	−0.01	0.03	0.09	0.02	−0.02	−0.39
	3	0.08	0.02	0.27	0.11	−0.22	0.03
	6	−0.19	−0.36	−0.23	−0.11	−0.18	−0.25
Vitamin B_12_ (µg)	1	0.21	0.08	0.03	0.15	0.06	0.02
	3	0.10	−0.01	0.16	0.13	−0.29	0.02
	6	0.14	−0.09	−0.08	0.18	−0.29	0.15
Folic acid (µg)	1	0.11	−0.02	0.04	0.16	−0.05	−0.06
	3	0.19	0.02	0.30	0.21	−0.24	0.12
	6	−0.36	−0.07	−0.12	−0.40	0.02	−0.41

^1^ Energy is presented as kilocalories (kcal) per 100 mL. ^2^ Macronutrients and dry matter are presented as grams per 100 mL. Data are presented as Pearson’s r coefficients.

**Table 6:** Correlations between human milk composition and mother’s body composition.

Mothers’ Body Composition	Month of Lactation	Composition of Human Milk					
		Energy ^1^	Total Protein ^2^	True Protein ^2^	Fat ^2^	Carbohydrates ^2^	Dry Matter ^2^
Weight (kg)	1	0.32 *	0.21	0.18	0.30	0.21	0.52 *
	3	0.43 *	0.63 *	0.51 *	0.37	0.30	0.49 *
	6	0.41	−0.14	−0.21	0.49 *	0.30	0.42
Body mass index (kg/m^2^)	1	0.33 *	0.27	0.24	0.33 *	0.20	0.48 *
	3	0.39 *	0.59 *	0.45 *	0.35	0.24	0.45 *
	6	0.44	0.01	−0.05	0.52	0.34	0.45
Fat mass (kg)	1	0.17	0.19	0.14	0.15	0.14	0.33 *
	3	0.42	0.64 *	0.51 *	0.36	0.29	0.48 *
	6	0.43	−0.10	−0.12	0.51	0.24	0.43
Fat mass (%)	1	0.32 *	0.26	0.23	0.29	0.17	0.46 *
	3	0.39	0.60 *	0.47 *	0.33	0.32	0.46 *
	6	0.42	0.01	−0.01	0.48	0.22	0.41
Fat free mass (kg)	1	0.24	0.10	0.06	0.22	0.25	0.52 *
	3	0.37	0.50 *	0.42	0.32	0.29	0.43 *
	6	0.15	−0.34	−0.38	0.22	0.06	0.17
Total body water (L)	1	0.27	0.19	0.15	0.27	0.10	0.49 *
	3	0.35	0.54 *	0.42	0.30	0.27	0.41
	6	0.25	−0.33	−0.38	0.31	0.13	0.26
Total body water (%)	1	−0.27	−0.20	−0.20	−0.23	−0.22	−0.41 *
	3	−0.46 *	−0.60 *	−0.52 *	−0.40	−0.35	−0.52 *
	6	−0.38	−0.06	−0.06	−0.44	−0.17	−0.37
Protein (kg)	1	−0.09	−0.10	−0.07	−0.14	0.12	−0.01
	3	0.16	0.05	0.11	0.14	0.14	0.15
	6	−0.22	−0.21	−0.19	−0.19	−0.21	−0.19
Muscles (kg)	1	0.26	0.10	0.04	0.22	0.28	0.53 *
	3	0.44 *	0.47 *	0.40	0.39	0.37	0.50 *
	6	0.03	−0.10	−0.12	0.08	0.10	0.13

^1^ Energy is presented as kilocalories (kcal) per 100 mL. ^2^ Macronutrients and dry matter are presented as grams per 100 mL. Data are presented as Pearson’s r coefficients. * *p* < 0.05.
